# CD34 Antibody-Coated Biodegradable Fiber Membrane Effectively Corrects Atrial Septal Defect (ASD) by Promoting Endothelialization

**DOI:** 10.3390/polym15010108

**Published:** 2022-12-27

**Authors:** Bin Chu, Xiaoli Li, Shiqiang Fan, Jinmei He, Xiaohong Ge, Hui Li, Changsheng Chen, Zhen Wang, Song Wang, Boning Li

**Affiliations:** 1 School of Materials Science and Engineering, Xiamen University of Technology, Xiamen 361024, China; 2 Key Laboratory of Biomedical Materials and Implant Devices, Research Institute, Tsinghua University, Shenzhen 518057, China; 3 Pediatric Cardiology, Shenzhen Children’s Hospital, Shenzhen 518057, China

**Keywords:** biodegradable, CD34 antibody, endothelialization, fiber membrane

## Abstract

Biodegradable materials are a next-generation invention for the treatment of congenital heart diseases. However, the corresponding technology used to develop ideal biomaterials still presents challenges. We previously reported the first biodegradable atrial septal defect (ASD) occluder made of poly-lactic acid (PLLA). Unfortunately, the PLLA occluder had a limited endothelialization effect. In this study, the surface of the occluder membrane was coated with sericin/CD34 antibodies to promote the growth of endothelial cells and the regeneration of defective tissue and enhance the repair of the atrial septal defect. The physicochemical properties of the coat on the surface of the fiber membrane were characterized. The sericin coat successfully covered the fiber surface of the membrane, and the thickness of the membrane increased with the sericin concentration. The swelling rate reached 230%. The microscopic observation of fluorescently labeled CD34 antibodies showed that the antibodies successfully attached to the fiber membrane; the fluorescence intensity of PLLA-SH5 was particularly high. The in vitro experiment showed that the PLLA-SH-CD34 fiber membrane was biocompatible and promoted the adhesion and proliferation of endothelial cells. According to our findings, the PLLA-SH-CD34 membrane provides a theoretical and technical basis for the research and development of novel biodegradable occluders.

## 1. Introduction

Congenital heart disease is a disorder or structural abnormality of the heart or large blood vessels that seriously affects the quality of life of patients [1]. Many procedures have been developed to correct structural heart defects [2]. These procedures are safe; therefore, they are the preferred treatment for congenital heart disease.

**Figure 1:**
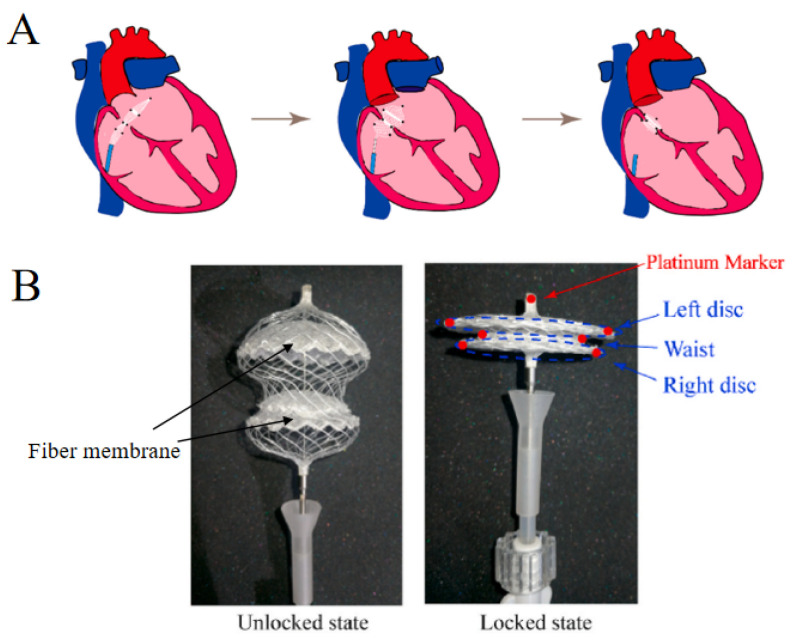
Preparation of a biodegradable ASD occluder (**A**) and photos of ASD in different states (**B**).

Currently, most cardiac occluders are prepared from super-elastic nickel-titanium alloy wires and are self-expanding, double-umbrella structures placed at the heart defect site [3]. For the treatment of congenital heart disease, ventricular septal defects, and atrial septal defects, the aim is to achieve complete closure of the heart defect. Although this successfully corrects the heart defect, given the fact that the occluder is a non-biodegradable material, it remains in the body permanently. To address this issue, we corrected early-stage atrial septal defect (ASD) in children using biodegradable polyester (PLLA) (Figure 1) [4]. The children had not developed any complications at two years follow-up. Despite this, the degradable PLLA still needs further improvement. The degradation of PLLA is related to its adaptation to surrounding cells, toward which the surface treatment of biomaterials represents an important way to improve material properties [5].

Cardiovascular occluders that are currently on the market are all non-degradable materials which exist permanently when implanted in the body; therefore, foreign body rejection may occur. If these occluders were made of degradable materials, they could seal the defect while promoting the regeneration and repair of surrounding tissues. After the repair is complete, they could degrade into small molecules and water to be absorbed by the body, which represents a more ideal treatment method [6,7]. Several biodegradable aliphatic medical polyesters have been developed [8]. Polylactide (PLA) degrades into carbon dioxide and water in the body and was approved for clinical use in 1997 by the Food and Drug Administration (FDA) [9]. PLA has been widely used in different medical devices, and its analogs, such as poly (lactic acid-co-gly-col) and other copolymers, have also been studied [9,10,11]. For example, the surface modification of electrospun PLA fiber mats with naturally occurring l-ascorbic acid (ASA) and fumaric acid (FA) enhanced the wettability of material and its antibacterial properties against Gram-positive *Staphylococcus aureus* and Gram-negative *Escherichia coli* [12]. Surface-modified PLA orthopedic screws with poly (vinyl alcohol) (PVA)-nanohydroxyapatite (nHA) nanofibers were shown to enhance the adhesion and biocompatibility of screws with osteoblast MC3T3-E1 cells [13]. In another study, the facile and versatile superhydrophilic coating of PLA via the stepwise deposition of metal/phenolic networks (MPNs) was carried out using a layer-by-layer assembly technique to mimic endothelium function. The optimized coating significantly inhibited the adhesion and activation of platelets, selectively supported the adhesion of endothelial cells, and suppressed the proliferation of smooth muscle cells [14]. In new congenital heart defects, the surface of the occluder should have good biocompatibility to promote the growth and proliferation of endothelial cells [15] and the regeneration of defective tissue when the material degrades to achieve the desired effect [16].

In the present study, a biodegradable plugging agent was prepared using polylactic acid (PLLA). Experiments showed that PLLA has good processing and mechanical properties [4]. Figure 1 shows the PLA ASD that was designed and developed in the present study. The membrane in contact with the inner wall of the heart on both sides of the occluder was woven with finer PLLA fibers. The present research focused on a fiber membrane (PLLA non-woven fabric) that was covered with a functional coat to promote endothelialization. The novel findings of this study provide a solid foundation for developing next-generation degradable materials that promote endothelialization.

## 2. Materials and Methods

### 2.1. Purification of Sericin

A total of 9.2 g of natural sericin powder (SP, Yi Xian Raw Silk Factory, Suzhou, China) was added to 250 mL of deionized water. The mixture was stirred with a magnetic stirrer, boiled for 15 min, and suction-filtered to obtain sericin, which was dissolved in boiling water to remove insoluble impurities. The filtration was repeated twice, and the sericin was freeze-dried and stored at room temperature until further use.

### 2.2. Preparation of the Sericin-Modified PLLA Membrane (PLLA-NH2)

**Scheme 1:**
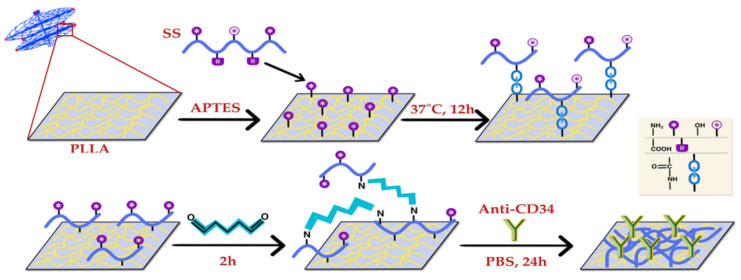
Schematic diagram of CD34 antibody coating of the fiber membrane surface.

The schematic diagram of CD34 Antibody-Coated membrane is shown in Scheme 1. The PLLA membrane (Xianjian, Shenzhen, China) was soaked in 2 mg/mL ATPES (3-aminopropyltrioxysilane, Sigma, St. Louis, MO, USA) anhydrous alcohol solution for 4 h at 37 °C under constant stirring. The membrane was then rinsed and soaked in 0.01 M HCL (Aladdin, Shanghai, China) solution for 3 h. Then, the membrane was rinsed again and dried in a vacuum. PBS solutions with concentrations of 1 mg/mL, 3 mg/mL, 5 mg/mL, and 10 mg/mL sericin (pH = 8.0) were prepared by dissolving an appropriate amount of sericin in PBS at 100 °C for 30 min and cooled until further use. EDC (Sigma, St. Louis, MO, USA) and NHS (Sigma, St. Louis, MO, USA) were added to the sericin solution at a ratio of 2:2:3. The solution was stirred for 30 min at room temperature. The PLLA membrane was then soaked in the sericin solution, stirred for 3 h at room temperature, and later incubated at 37 °C for 12 h under constant shaking at 100 rpm. The membrane was then rinsed, freeze-dried, and stored until further use. Finally, the PLLA membrane was soaked for 2 h in a glutaraldehyde (Aladdin, Shanghai, China) solution containing 0.25% PBS and thereafter washed thoroughly with water for 3 days. The water was changed three times per day. The membrane was then freeze-dried at −80 °C for later use.

### 2.3. Preparation of the CD34 Antibody-Modified PLLA Membrane

A CD34 antibody (Abcam, Cambridge, UK) solution with a concentration of 4 μg/mL in sterile PBS (pH = 7.4) was prepared. The sericin-modified PLLA membrane was soaked in the CD34 antibody solution and incubated overnight to obtain the modified PLLA endothelialization membrane.

### 2.4. Scanning Electron Microscopy (SEM) Analysis

The materials were freeze-dried and sputter-coated with a conductive layer of gold to enhance surface conductivity. The membrane was then observed using a scanning electron microscope (MIRA3, Tescan, Brno, Czech) at a voltage of 10 kV and a current of 10 mA.

### 2.5. Contact Angle Measurement

A digital contact angle measurement system with a CCD camera (DSA-100, KRUSS, Hamburg, Germany) was used to measure the water contact angles of the fiber membranes using the sessile drop method. A piece (1 cm × 1 cm) of the membrane was attached to the glass slide with double-faced adhesive tape to remain intact during the wetting process. Pure water droplets (9 μL) were then dripped onto the surface of the sample. Temporal images of the system were captured and analyzed at room temperature. The contact angle was measured and calculated using simulation software. The measurement was repeated 3–5 times for at least three samples.

### 2.6. Water Swelling Rate

The fiber membranes were soaked in ultrapure water at room temperature to assess their swelling capacity. The swollen membranes were then weighed, and the swelling index was determined based on the change in weight at different times. The analysis was performed in triplicate, and the swelling index (%) was calculated as follows:

Swelling index = (Mw − Ma)/Ma × 100%

where Ma is the dry weight of the hydrogels and Mw is the weight of the hydrogels after swelling.

### 2.7. Mechanical Property Test

Stress–strain curves were recorded using an Instron electronic universal testing machine (Instron Corporation, Norwood, MA, USA) at a crosshead speed of 0.5 mm/min. The measurement was performed using 5 cm × 1.5 cm × 0.2 cm membranes. At least five samples were measured to enhance the reliability of the readings.

### 2.8. FT-IR

The secondary structure of the membranes was determined using an FT-IR spectrophotometer (Nicolet-iS50, Thermo, Waltham, MA, USA). Freeze-dried membranes were placed onto a “golden gate” diamond window and examined at a spectral region of 500–4000 cm^−1^.

### 2.9. XPS

A 5 × 5 mm sample of the membrane was attached to a sample tray and transferred to an XPS sample room (Scientific K-Alpha, Thermo, Waltham, MA, USA). When the pressure in the sample room reached less than 2.0 × 10^−7^ mbar, the membrane was transferred to the analysis room for observation using SEM at a spot size of 400 μm, a working voltage of 12 kV, and a filament current of 6 mA. The full-spectrum scanning energy was 150 eV, and the step size was 1 eV. Narrow-spectrum scanning had a pass energy of 50 eV and a step size of 0.1 eV.

### 2.10. Cytocompatibility of Fiber Membranes

A cytocompatibility analysis of the modified fiber membranes was performed using human umbilical vein endothelial cells (HUVECs) (iCell Bioscience Inc., Shanghai, China). HUVECs were obtained from Hunan Fenghui Biotechnology Co., Ltd., Changsha, China. The membranes were cast in 48-well chamber slides (n = 5) and sterilized with UV radiation for 24 h. Endothelial cells (1 × 10^4^) that were seeded onto each gel in tissue culture-treated plates were used as a control. The cells were cultured at 37 °C under 5% CO_2_, and the medium was changed every two days. A cell counting kit-8 (CCK-8) was used to assess the proliferation of the cells on the membranes. The cell culture medium was discarded on day 7, and the cells were rinsed twice with sterile PBS (pH 7.4). Thereafter, 200 μL of fresh DMEM that contained 10% v/v CCK-8 reagent (Cell Counting Kit-8, Dojindo Laboratorise, Shanghai, China) was added to each well. The cells were then incubated at 37 °C for 3 h. Afterwards, 100 μL of media from each well was transferred to a 96-well plate. The absorbance of the 96-well plate was read at 450 nm using a microplate reader (BIO-RAD, Hercules, CA, USA). For live/dead assays, the cells were stained with calcein-AM and PI (Calcein-AM/PI Double Staining Kit, Dojindo Laboratorise, Shanghai, China), and the numbers of live (green) and dead (propidium iodide-stained) cells were counted under a confocal microscope.

### 2.11. Statistical Analysis

The data were analyzed using SPSS software. Continuous, normally distributed variables were presented as the mean ± SD. Multiple groups were compared using one-way ANOVA. *p* < 0.05 was considered statistically significant.

## 3. Results

### 3.1. Characteristics of Sericin-Modified Fiber Membranes

**Figure 2:**
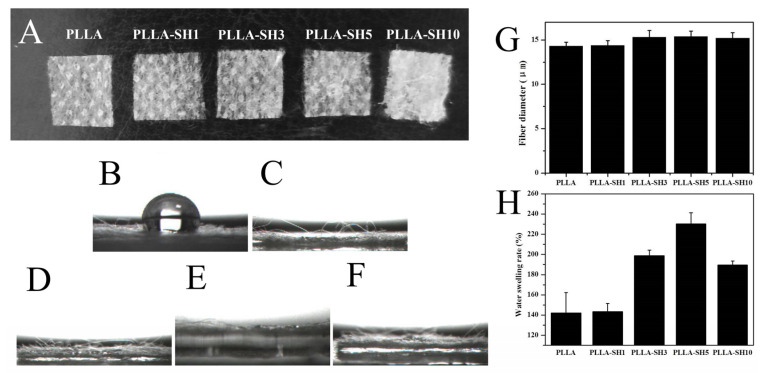
Characteristics of the sericin-modified fiber membranes: (**A**) photos of PLLA, PLLA-SH1, PLLA-SH3, PLLA-SH5, and PLLA-SH10; (**B**–**F**) contact angle analysis of PLLA, PLLA-SH1, PLLA-SH3, PLLA-SH5, and PLLA-SH10; (**G**) fiber diameter; and (**H**) swelling rate.

**Figure 3:**
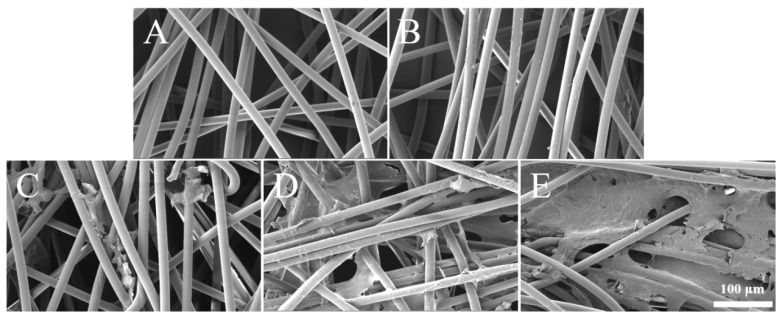
SEM images of PLLA (**A**), PLLA-SH1 (**B**), PLLA-SH3 (**C**), PLLA-SH5 (**D**), and PLLA-SH10 (**E**).

The characteristics of the sericin-modified fiber membranes are shown in Figure 2 and Figure 3. The thickness of the coat formed on the surface of the PLLA membrane increased with the sericin concentration. At 10 mg/mL of sericin solution, a thick coating formed on the surface of the PLLA membrane, and this coating was unstable and peeled off easily. For sericin concentrations lower than 5 mg/mL, the thickness of the membrane was moderate. The contact angle analysis of the sericin-modified PLLA film showed that the contact angle of the PLLA barrier film was 101° and the contact angle of the sericin-coated barrier film was 0°. This was due to the fact that the surface of sericin is rich in amino groups and carboxyl groups which greatly enhance the hydrophilic properties of the fiber surface. Therefore, the tension of droplets on the membrane surface was very small, and they flowed out from the fiber gap. No liquid was observed on the surface of the membrane. Studies have shown that the hydrophilic and hydrophobic properties of the surface of a material have a considerable impact on cell adhesion and growth [17] as hydrophilic surfaces are more conducive to the binding of the CD34 antibody, thereby exerting a corresponding effect on endothelial cells [18].

Changes in the fiber diameters of the PLLA and PLLA coatings were also investigated. As shown in Figure 2G, the diameter of the fibers in the PLLA membranes partially increased with the sericin concentration, although this change was not statistically significant. The water absorption and swelling rate of the membrane are shown in Figure 2H, which illustrates that the water absorption and swelling rate of the film increased with the sericin concentration. The swelling rate peaked at 5 mg/mL sericin solution with a value higher than 230%. The hydrophilicity and hydrophobicity of the membrane influenced the adhesion of cells. The hydrophilic and swelling properties of the modified PLLA membrane were greatly enhanced, which provided a conducive environment for the adhesion and growth of endothelial cells.

The modified fiber membrane was also observed using SEM. As shown in Figure 3, an increase in the concentration of sericin had no effect on the fiber structure, although more and more observable fibers appeared between the existing fibers. Part of the sericin bound tightly to the PLLA fibers, which indicated the successful preparation of the composite. Considering that the 10% concentration was not suitable, 1%, 3% and 5% concentrations were selected for characterization.

### 3.2. Mechanical Property Analysis

**Figure 4:**
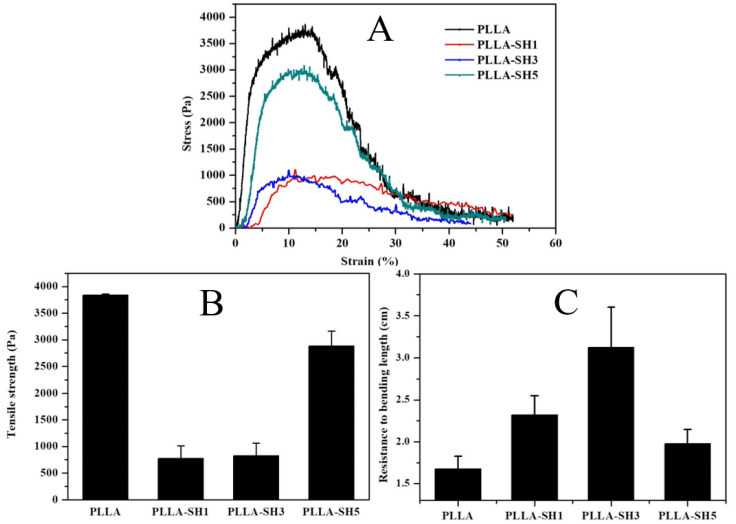
Characteristics of the sericin-modified fiber membranes. (**A**) Tensile stress–strain curves of the fiber membranes. (**B**) Tensile strength of the fiber membranes. (**C**) Fabric bending stiffness of the modified PLLA membrane.

Occluder fiber membranes for clinical applications should have a certain strength. Appropriate mechanical properties are conducive to the transportation and application of the occluder in the body and ensure the integrity of the membrane to block blood flow. There are many evaluation methods used to characterize these materials [19]. In this study, we used the tensile strength test. As shown in Figure 4A,B, the tensile strength of the PLLA membrane decreased after sericin modification, which could be due to the addition of ATPES and HCL affecting the crystallization properties and strength of PLLA. The tensile strength of the membrane gradually increased with the increase in the sericin concentration. The breaking strength was highest in PLLA-SH5 and was comparable to that of unmodified PLLA.

The fabric bending stiffness of the modified PLLA membrane was analyzed using the inclined plane method. As shown in Figure 4C, the bending stiffness of the modified membrane increased, and the corresponding bending radii of PLLA, PLLA-SH1, PLLA-SH3, and PLLA-SH5 were 1.68 ± 0.19, 2.27 ± 0.28, 3.08 ± 0.49, and 1.93 ± 0.18 cm, respectively. Considering the tensile strength results, the PLLA-SH5 group was selected for subsequent CD34 antibody modification.

### 3.3. Characterization of PLLA, PLLA-NH2, and PLLA-SH5

**Figure 5:**
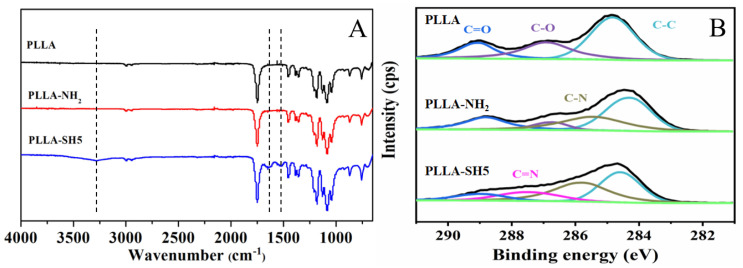
Characteristics of the modified PLLA membrane. (**A**) FT-IR spectrogram and (**B**) XPS.

The infrared absorption spectra of PLLA, PLLA-NH2, and PLLA-SH5 are shown in Figure 5. The absorption peak of amide I for sericin occurred at 1615 cm^−1^, which was indicative of a β-sheet structure. The absorption peak of amide II occurred at 1521 cm^−1^, which was indicative of a β-sheet folded structure, while the absorption peak of amide III occurred at 1240 cm^−1^, which was indicative of an α-random coil structure [20]. Compared with PLLA and PLLA-NH2, PLLA-SH5 had the characteristic absorption peaks of amide I and II of sericin at 1615 cm^−1^ and 1521 cm^−1^. This indicated that sericin was attached to the surface of the PLLA fiber membrane in the PLLA-SH5 material. Thus, the sericin-modified PLLA membrane was prepared successfully.

The XPS bond energy data in Figure 5B show that compared with the PLLA sample membrane, the peak of CN at 285.8 eV in PLLA-NH2 confirmed the successful amination of PLLA, while in PLLA-SH5, the C = N peak was observed at 287.5 eV. The presence of C = N demonstrated the existence of glutaraldehyde cross-linking. The elemental analysis and comparison (Table 1) based on the significant increase in nitrogen showed that sericin was successfully immobilized on the surface of the PLLA membrane.

PLLA-NH2 and PLLA were then compared. The N content was higher in PLLA, which indicated that the surface of the PLLA membrane was successfully aminated. The N content was lower in PLLA-SH5 than in PLLA-NH2 because the amino group on the surface of PLLA-NH2 was in the sericin solution. The carboxyl group of the carboxylate underwent a Schiff base reaction to fix the sericin on the surface of the material, which reduced the N content. These results are consistent with the FT-IR results.

**Figure 6:**
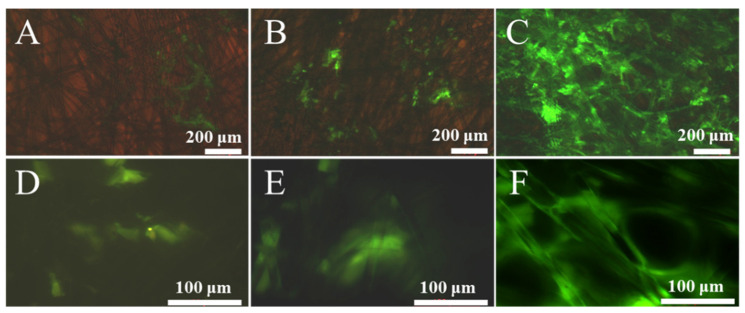
The appearance of PLLA-SH1 (**A**,**D**), PLLA-SH3 (**B**,**E**), and PLLA-SH5 (**C**,**F**) under a fluorescence microscope.

PLLA-SH-CD34 was prepared by coating PLLA-NH5 with CD34 antibodies. FITC-fluorescently labeled CD34 antibodies were then observed to indicate the binding of the material. As shown in Figure 6, the fluorescence intensity of PLLA-SH1, PLLA-SH3, and PLLA-SH5 gradually increased with the sericin concentration. Moreover, a large green area was observed in PLLA-SH5, which indicated an increase in CD34 antibodies that were immobilized on the coat surface. Based on our findings, and in combination with results from previous studies, PLLA-SH5 was selected for CD34 antibody coating and the subsequent experiments.

### 3.4. Cytocompatibility

**Figure 7:**
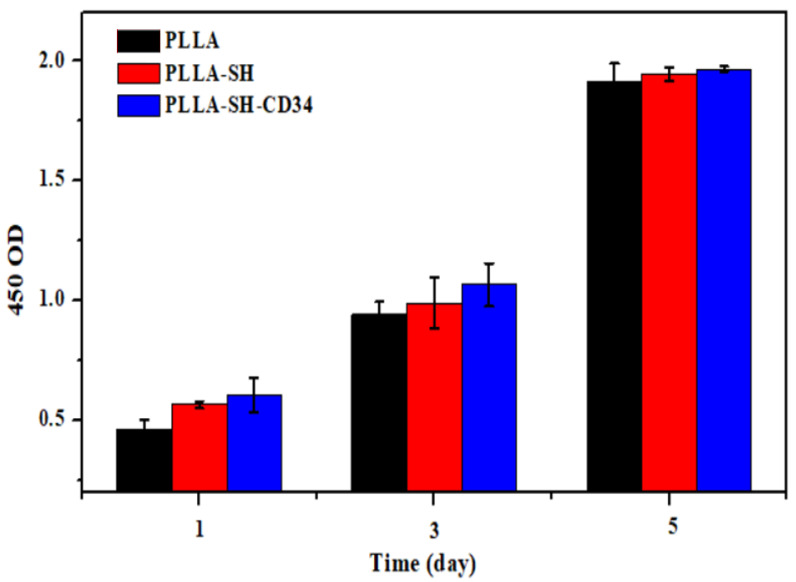
Proliferation of HUVECs on the fiber membranes. CCK-8 assay results for L929 cells on different scaffolds on days 1, 3, and 5. N = 3.

Figure 7 shows the OD values of the PLLA, PLLA-SH5, and PLLA-SH-CD34 surfaces cultured for 1, 3, and 5 days. OD was positively correlated with the cell proliferation rate. The proliferation of the endothelial cells on the surface of the membrane in the three groups gradually increased with time. The cell proliferation was slightly higher on PLLA-SH and PLLA-SH-CD34 than on pure PLLA, which indicated that the coating had better biocompatibility and less cytotoxicity toward PLLA.

**Figure 8:**
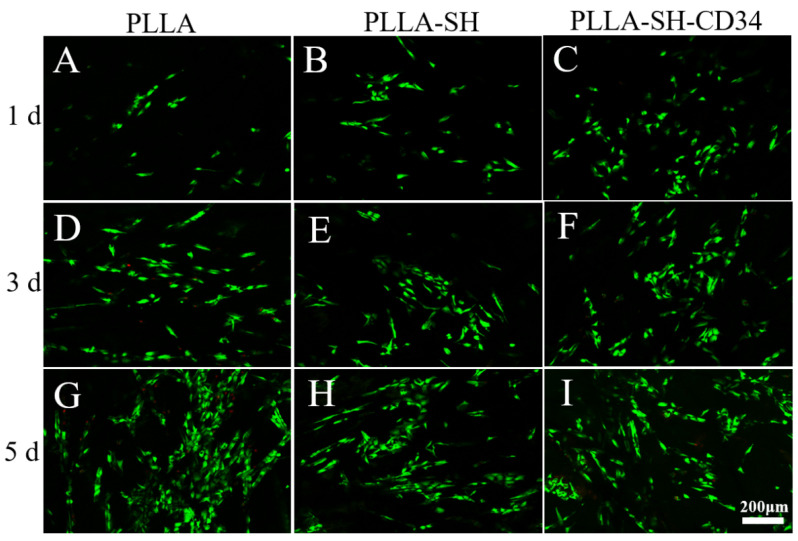
Live/dead staining of endothelial cells on different scaffolds on days 1, 3, and 5.

**Figure 9:**
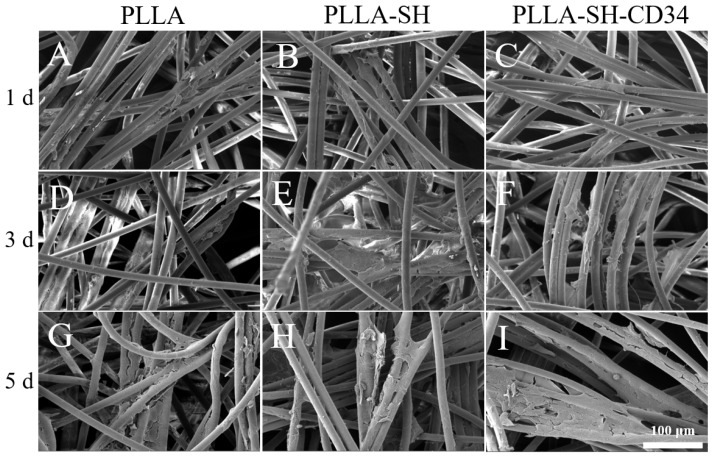
SEM images of endothelial cells on different scaffolds on days 1, 3, and 5.

The images of the laser confocal and SEM observations of live and dead cells stained in the three groups of materials on days 1, 3, and 5 are shown in Figure 8 and Figure 9. The rate of cell proliferation was consistent with the cytocompatibility results. The proliferation of the endothelial cells gradually increased with time. The cells grew on the surface of the membrane fibers or gradually filled in the gaps between the fibers. No dead (red) cells were observed using laser confocal microscopy because the dead cells could not adhere to the three-dimensional membrane. SEM showed that the morphology of cells that grew in gaps that were between and on the surface of the fibers was normal. The endothelial cells adhered and proliferated on the surfaces of the three membranes and were higher in the PLLA-SH-CD34 group. A similar trend was observed for cell attachment (Figure 9). The physical and chemical properties of a material have a certain influence on the adhesion of surface cells. A dense network structure, suitable hydrophilicity and hydrophobicity, and the induction of factors promote the adhesion and proliferation of endothelial cells [21,22]. Therefore, PLLA-SH-CD34 showed the best performance.

**Figure 10:**
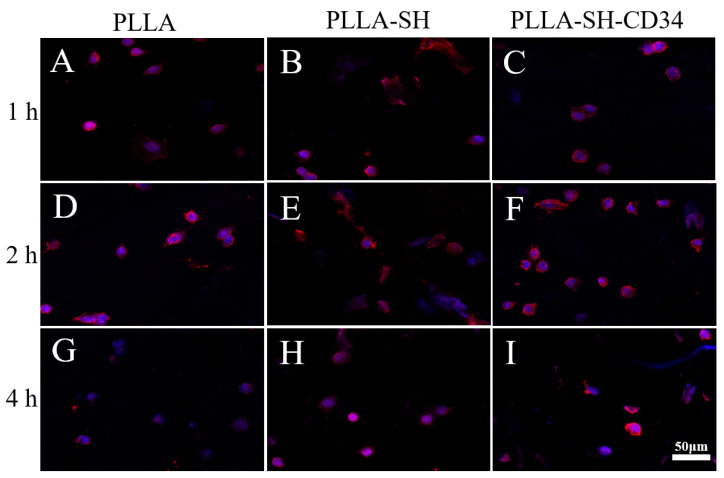
Growth of endothelial cells on different scaffolds at different times. The analysis was performed using phalloidin and nuclear staining.

Nuclei staining and an adherence analysis of the cells on the fibrous membrane surface were performed at 1 h, 2 h, and 4 h. As shown in Figure 10, the adhesion of the cells on the fiber membrane surface changed with time as the cells spread out gradually. Compared with the rest of the materials, the red fluorescence outside the nucleus of the endothelial cells on PLLA-SH-CD34 was relatively bright after 4 h, and the cells were more spindle-shaped, which suggested that they were suitable for growth. These findings indicated that the PLLA-SH-CD34 membranes promoted the adhesion and growth of the endothelial cells.

## 4. Discussion

Existing occluders are made of various materials, such as titanium, cobalt chromium, polyethylene, polytetrafluoroethylene, etc. These materials will not cause a host immune response and inflammation; however, blood compatibility is a problem. Even if degradable biomaterials, such as PLLA, are used to replace the original non-degradable materials, the establishment of a monolayer of endothelial cells (ECs) on the blood-contact surface is considered the most effective way to improve blood compatibility and product performance issues [6].

Endothelialization is an unavoidable function [23]. The endothelial layer, which is the interface that is in direct contact with blood, participates in maintaining vascular homeostasis, signal transfer, and metabolic exchange and preventing thrombosis [24].

Surface engineering has generated new biomaterials that promote re-endothelialization after implantation in the body [25]. In 1997, Asahara et al. discovered endothelial progenitor cells (EPCs) in peripheral blood and demonstrated their important role in endothelialization [26]. This led to the rapid development of techniques and procedures that utilize EPCs, for example, in the repair of the endothelial layer. EPCs migrate from the bone marrow to the damaged part of the blood vessel and rapidly differentiate and proliferate into ECs, which repairs the damaged area by forming a new endothelial layer [27]. The discovery of the differentiation of EPCs set the stage for further studies, including some that immobilized EPC-specific recognition molecules, such as CD34 antibodies, on the surface of scaffolds to capture and recruit EPCs for in situ endothelialization [28]. More research is now focused on how to prepare endothelial cell layers on the surface of cardiovascular devices [29,30]. Li [31] designed a series of sol–gel coatings loaded with an inhibitor on ZE21B Mg alloy to improve corrosion resistance and endothelialization, aiming at a potential cardiovascular application. The results suggested the potential of a Schiff base inhibitor-loaded sol–gel coating for enhanced corrosion protection and biocompatibility in bioabsorbable cardiovascular implants. Kamil [32] developed a polydopamine and gelatin coating for the rapid endothelialization of vascular scaffolds. The coating significantly enhanced endothelialization on flat surfaces, tubular small-diameter scaffolds, and commercial vascular prostheses.

In the present study, we improved the wettability of PLLA membranes by using surface treatment. The membrane was also coated with bioactive molecules and CD34 antibodies to promote the adhesion and proliferation of ECs and accelerate endothelialization. Although the spread of Ecs on the surface of the biomaterial significantly improved, the coverage rate was still sub-optimal, and endothelialization was still slow. In the future, we will conduct more in-depth animal experiments to verify its effectiveness. Therefore, research on how to improve re-endothelialization and materials that can support this process and its necessary technologies is still required.

## 5. Conclusions

In conclusion, we developed a sericin/CD34 antibody-coated fibrous occluder membrane that can promote the growth of endothelial cells and enhance the regeneration of defective tissue in the heart. The sericin-modified coating successfully attached to the surface of the fiber membrane, which was confirmed by electron microscope observation and component analysis. The hydrophilic properties of the modified PLLA membranes greatly improved, and the successful CD34 antibody modification of the membrane was confirmed by fluorescence microscopy. In vitro experiments revealed that the fibrous membrane coated with CD34 antibodies was highly biocompatible, which promoted the adhesion and proliferation of endothelial cells. The cells in the PLLA-SH-CD34 group showed more of a spindle shape that was suitable for growth. The findings of this study provide a theoretical and technical basis for the research and development of next-generation biodegradable occluders.

## Figures and Tables

**Table 1:** XPS results of the atomic composition of PLLA, PLLA-NH2, and PLLA-SH5.

Samples	C (%)	O (%)	N (%)
PLLA	67.8	30.38	1.83
PLLA-NH_2_	68.04	20.38	11.58
PLLA-SH5	67.24	24.23	8.53

## Data Availability

Not applicable.
